# Non-Pharmacological and Non-Optical Interventions for Myopia Prevention and Control in Children: A Systematic Review and Meta-Analysis

**DOI:** 10.3390/children13070915

**Published:** 2026-07-10

**Authors:** Claudia Domínguez-Morras, Miren Paniagua García, Isabel Castro Garrido, Javier Moreno-Montañés, Alejandro Fernandez-Montero, Laura Moreno-Galarraga

**Affiliations:** 1 Faculty of Science, Universidad Pública de Navarra, UPNA, 31008 Pamplona, Spain; 2 Department of Pediatrics, Hospital Universitario de Navarra, HUN, 31008 Pamplona, Spain; 3 Department of Ophthalmology, Hospital Fundación Jiménez Díaz, 28040 Madrid, Spain; drmorenomontanes@gmail.es; 4 Instituto de Investigación Sanitaria de Navarra, IdiSNA, 31008 Pamplona, Spain; 5 Department of Occupational Medicine, Universidad de Navarra, 31008 Pamplona, Spain

**Keywords:** myopia, prevention and control, low-level light therapy, photobiomodulation, dietary supplements, axial length, spherical equivalent, children

## Abstract

**Highlights:**

**What are the main findings?**

- Repeated low-level red-light therapy (RLRL) is associated with reduced axial elongation and slower myopia progression in children, although substantial heterogeneity was observed across studies.
- Current evidence is insufficient to demonstrate a consistent clinically meaningful effect of nutritional supplementation on axial elongation or myopia progression.

**What is the implication of the main finding?**

- RLRL represents a promising non-pharmacological strategy for myopia control, but further studies with longer follow-up and more diverse populations are needed.
- The current evidence does not support recommending nutritional supplementation as an effective strategy for myopia prevention or control in children.

**Abstract:**

Background: Myopia is projected to affect 50% of the global population by 2050. Its progression increases the risk of severe ocular pathologies, making it imperative to validate safe and effective non-pharmacological prevention interventions. Aim: To evaluate the clinical efficacy of low-level red-light therapy (RLRL) and nutritional supplementation in preventing and controlling myopia progression in children and adolescents. Methods: This systematic review and meta-analysis followed PRISMA guidelines and was registered in PROSPERO (CRD420261301596). Randomized clinical trials in pediatric populations (aged 0–18 years) were included. The primary quantitative outcome measures for myopia were axial length (AL) and spherical equivalent (SE). Risk of bias (RoB 2) and heterogeneity (I^2^) were assessed. Results: 18 articles were included (*n* = 2438). RLRL demonstrated a significant reduction in axial elongation (mean difference (MD): −0.26 mm; 95% CI: −0.34 to −0.18) and a protective effect on refractive progression (MD: +0.60 D; 95% CI: 0.42 to 0.78), although with high heterogeneity (I^2^ > 97%). Nutritional supplementation exhibited a modest, non-significant effect on axial length (−0.22 mm; 95% CI: −0.44 to 0.00), acting primarily as functional support. Conclusions: RLRL slows ocular elongation and myopia progression. Recent studies provide reassuring evidence regarding its long-term safety when recommended treatment protocols are followed. However, the high heterogeneity among studies, the need for standardized treatment discontinuation strategies, and the limited long-term evidence in geographically and ethnically diverse pediatric populations warrant further investigation. Current evidence is insufficient to demonstrate a consistent clinically meaningful effect of nutritional supplementation on axial elongation.

## 1. Introduction

Myopia is a type of ametropia in which the eye exhibits an increased axial length or an excessive optical power for accommodative spasm, corneal diseases or nuclear cataract. In myopic eyes, the refracted light is focused in front of the retina instead of onto the retina, resulting in blurred distance vision [1]. It is currently recognized as one of the most significant public health challenges of the 21st century. Epidemiological projections indicate an alarming upward trend, estimating that by 2050, myopia secondary to increased axial length will affect approximately 5 billion people (50% of the global population), with 1 billion (10%) suffering from high myopia [1,2]. The relevance of this condition lies not only in its epidemiological magnitude but also in its long-term pathological consequences. Excessive elongation of the eyeball exponentially increases the risk of severe, irreversible visual impairment due to comorbidities such as myopic maculopathy, rhegmatogenous retinal detachment, posterior subcapsular cataracts, and open-angle glaucoma [3]. Therefore, the identification and implementation of effective preventive strategies during the critical stages of childhood ocular development are essential to limit the progression and long-term complications associated with myopia.

Myopia typically develops during early or middle childhood (between 6 and 12 years of age), a period during which its progression is also most rapid [4]. Axial length (AL) is the most variable biometric factor and strongly correlates with the refractive state; consequently, longer eyes are significantly more prone to developing pathologic myopia in adulthood [5]. Therefore, restricting excessive ocular elongation is crucial to halt the onset and progression of the disease [6]. For this reason, the control of axial elongation is widely considered the most objective and reproducible outcome measure for evaluating myopia progression [7]. The current paradigm for myopia management has shifted from passive optical compensation with single-vision spectacles to active control of its progression, using axial length (AL) and spherical equivalent (SE) as the primary clinical biomarkers [7]. Current evidence-based myopia-control strategies include low-dose atropine, orthokeratology, dual-focus soft contact lenses and defocus incorporated multiple segments (DIMS) spectacles. Although these interventions have demonstrated efficacy, limitations related to cost, adherence, accessibility or local side effects (e.g., photophobia or allergic conjunctivitis), as well as the risk of a “rebound effect” upon treatment discontinuation, have stimulated interest in emerging non-pharmacological alternatives [7,8,9,10]. As a result, there is growing clinical interest in exploring safe, accessible and non-invasive alternative therapies.

Among emerging non-pharmacological interventions, repeated low-level red-light (RLRL) therapy and specific nutritional supplementation have recently garnered significant attention. RLRL involves ocular exposure to low-intensity red light (typically ~650 nm) administered in brief, daily sessions. The underlying biological mechanism is thought to involve increased choroidal blood flow induced by long-wavelength red light, thereby thickening the choroid, mitigating scleral hypoxia, and modulating ocular growth through mitochondrial stimulation via photobiomodulation [11,12]. Conversely, the specific role of diet and nutritional supplementation in myopia remains a subject of intense debate. While traditional research has focused on the protective proxy of vitamin D, recent hypotheses suggest that certain macronutrients and antioxidant micronutrients (such as omega-3 polyunsaturated fatty acids, lutein, or crocetin) might modulate the metabolic and oxidative signalling pathways that regulate axial length [13,14]. Although repeated low-level red-light therapy and nutritional supplementation differ substantially in their mechanisms of action and current level of clinical evidence, both represent emerging non-pharmacological and non-optical strategies proposed for myopia prevention and control. Therefore, they were evaluated within the same conceptual framework while analyzed separately throughout this review.

Despite the growing popularity of these interventions, critical knowledge gaps persist in the literature. For RLRL therapy, concerns regarding standardization of protocols, long-term retinal safety, and potential rebound effects remain unresolved. For nutritional supplementation, the structural prophylactic capacity to actively halt axial elongation is highly debated, with existing clinical trials yielding scattered and heterogeneous results, and most studies performed in adults. The lack of robust consensus in the pediatric population group has motivated this review, with the aim of broadening and deepening knowledge in this field.

The objective of this review is to evaluate the clinical effectiveness of non-pharmacological and non-optical interventions-specifically repeated low-level red-light therapy and nutritional supplementation- in preventing and controlling myopia progression in children and adolescents. For this purpose, a systematic review and meta-analysis were performed to synthesize the available evidence, determine the extent of this effectiveness, and identify possible limitations and areas for future research.

## 2. Materials and Methods

This systematic review and meta-analysis were carried out following the Preferred Reporting Items for Systematic Reviews and Meta-Analyses (PRISMA) 2020 guidelines (available at: https://www.prisma-statement.org/PRISMAStatement, accessed on 5 July 2026). The protocol was previously registered in the PROSPERO (International Prospective Register of Systematic Reviews) database (ID: CRD420261301596) on 11 February 2026. The search strategy, inclusion and exclusion criteria, methods of information extraction, and statistical analyses used in the quantitative synthesis were defined in the protocol. The PRISMA checklist is provided in Supplementary File S1 and the complete PROSPERO protocol is provided as Supplementary File S2.

The key questions for this study were defined using the PICO format, considering the following elements:

- Population (P): Children and adolescents (0–18 years). Patients could be non-myopic (for incidence analysis) or myopic (for progression analysis).
- Intervention (I): Non-pharmacological therapies for the prevention and control of myopia, specifically based on light therapy (Repeated Low-Level Red-Light) and nutritional dietary supplementation.
- Comparison (C): Inactive controls (including placebo, sham treatment, no intervention) or standard optical correction (single-vision spectacles).
- Outcomes (O): The primary outcomes were changes in spherical equivalent (SE) measured in diopters, axial length (AL) measured in millimetres, and myopia incidence. Additional outcomes included Best Corrected Visual Acuity (BVCA), safety and adverse events, treatment adherence, and subfoveal choroidal thickness (SFCT).

### 2.1. Literature Search Strategy

The literature search was conducted using PubMed, Scopus, Web of Science, and the Cochrane Central Register of Controlled Trials (CENTRAL) electronic databases (Table 1). The search strategy was intentionally designed to maximize sensitivity and identify all potentially relevant studies addressing non-pharmacological and non-optical interventions. Specific keywords related to the topic and modified to suit the characteristics of each database were included. Search functions such as MeSH terms, text words, Boolean operators (AND, OR, NOT), and truncated searches were used appropriately. The search was limited to articles published from the year 2000 onwards, with no additional geographical or language filters applied. Subsequently, article selection was performed in two phases: reading titles and abstracts and then reading the full text. Each phase was performed independently by two reviewers, and discrepancies were resolved by a third independent investigator.

### 2.2. Literature Inclusion and Exclusion Criteria

The inclusion criteria were (1) studies aimed at pediatric and adolescent patients (aged ≤ 18 years); (2) studies where patients received a non-pharmacological intervention involving light therapy or nutritional supplementation; (3) studies with an adequate control group; and (4) Randomized Controlled Trials (RCTs) and Controlled Clinical Trials (quasi-randomized trials).

The exclusion criteria were (1)studies involving patients with ocular pathologies other than refractive error or systemics diseases affecting ocular growth; (2) studies where patients used concomitant active myopia control interventions (such as atropine); (3) interventions based solely on behavioural modifications without specific devices, or relying on surgical/optical control strategies (e.g., orthokeratology); (4) mixed-age populations where pediatric data could not be extracted separately; (5) observational studies, case reports, animal studies, meta-analyses or conference abstracts lacking sufficient data; (6) unfinished studies or studies published only in abstract form; and (7) studies with and inadequate control groups or missing control groups.

### 2.3. Study Selection and Data Extraction

The removal of duplicate articles was completed using Zotero reference management software (Zotero Version 7.0.15; Corporation for Digital Scholarship, Vienna, VA, USA). Titles and abstracts were reviewed for eligibility criteria independently by two investigators (Castro-Garrido I. and Panigua-Garcia M.) using the Rayyan web application. (Rayyan Systems Inc., Cambridge, MA, USA). Discrepancies were resolved by consensus together with a third investigator (Moreno-Galarraga L.). Full-text copies of the identified studies were obtained, and a detailed review by the lead author (Dominguez-Morras C.) was performed.

Data extraction was performed using a standardized extraction form and independently verified by a second reviewer. Extracted variables included study design, participant characteristics, intervention details, follow-up duration, outcome measures, and safety data.The completed AMSTAR-2 (A MeaSurement Tool to Assess Systematic Reviews 2) checklist, a validated instrument for the critical appraisal of the methodological quality of systematic reviews, is provided as Supplementary File S3. A detailed assessment of the risk of bias of each included randomized controlled trial using the Cochrane RoB 2 tool, including domain-specific judgments and the rationale for the overall assessment, is available as Supplementary File S4. 

### 2.4. Information Synthesis

The main objective of this review was to evaluate the effectiveness of non-pharmacological interventions in the prevention and control of myopia. To this end, changes in two objective clinical parameters- spherical equivalent (SE) and axial length (AL)- were compared from baseline to the end of the follow-up period. The clinical gold standard for quantifying myopia is the refractive error expressed in diopters (D). In pediatric populations, the high activity of the ocular accommodative system can lead to “pseudomyopia”, potentially overestimating the refractive error by 0.6 D to 0.8 D. To ensure maximum reliability, the included studies utilized cycloplegic refraction, typically administered via 1% cyclopentolate, to paralyze the ciliary muscle and accurately measure the refractive state [7]. Furthermore, monitoring the physical growth of the eye is considered the most objective indicator of disease progression and treatment efficacy. Axial length (AL) represents the physical distance in millimetres (mm) from the corneal vertex to the retinal pigment epithelium. Unlike refraction, AL is an anatomical measure that does not depend on patient response or accommodation. The studies included in this review employed non-invasive high-precision biometry methods, such as partial coherence interferometry, optical low coherence reflectometry (OLCR), or optical coherence tomography (OCT). Additionally, subfoveal choroidal thickness (SFCT), measured via OCT, was analyzed as an early marker of therapeutic response, as choroidal thickening has been associated with positive responses to low-intensity red-light therapies [7,10].

Mean differences between the intervention and control groups were calculated for all outcomes. When studies directly reported the pre/post- intervention mean difference with its measure of dispersion, the data were extracted directly. In cases where the original studies provided the 95% confidence interval (CI) instead of the standard deviation (SD), the SD was calculated algebraically using standardized formulas. Specifically, standard deviations (SDs) were estimated from the reported 95% confidence intervals and sample sizes using standardized statistical formulas. Studies lacking sufficient data to estimate the treatment effect were excluded from the meta-analysis and reserved for qualitative analysis.

The analyses were performed according to the predefined protocol registered in PROSPERO. Random-effects models were selected a priori because of the expected clinical heterogeneity among studies. Where appropriate, studies with multiple intervention arms were handled according to standard Cochrane recommendations, and studies with insufficient information to estimate treatment effects were excluded from quantitative synthesis. Publication bias was assessed by visual inspection of funnel plots. Meta-analyses were conducted using random-effects models with Stata software (version 14.0; StataCorp, College Station, TX, USA) through the *metan* command. Pooled estimates were graphically represented using forest plots displaying individual study effect sizes, confidence intervals, and overall combined effects. Heterogeneity was assessed using the I^2^ statistic, with values >50% considered indicative of substantial heterogeneity. Publication bias was assessed by visual inspection of funnel plots, as prespecified in the registered PROSPERO protocol. Formal statistical tests for funnel plot asymmetry (e.g., Egger’s regression) were not performed because they were not prespecified and are generally not recommended when fewer than 10 studies are available. Furthermore, the substantial clinical and statistical heterogeneity observed across studies would limit the interpretation of these tests [15]. Finally, the methodological quality was independently assessed using the AMSTAR 2 tool (2017). The complete evaluation is provided in the Supplementary Materials.

## 3. Results

A total of 880 potentially relevant records were identified. After removal of 186 duplicates, 474 records were excluded during the initial screening because they did not meet the required study design criteria (RCTs or CCTs).

**Figure 1:**
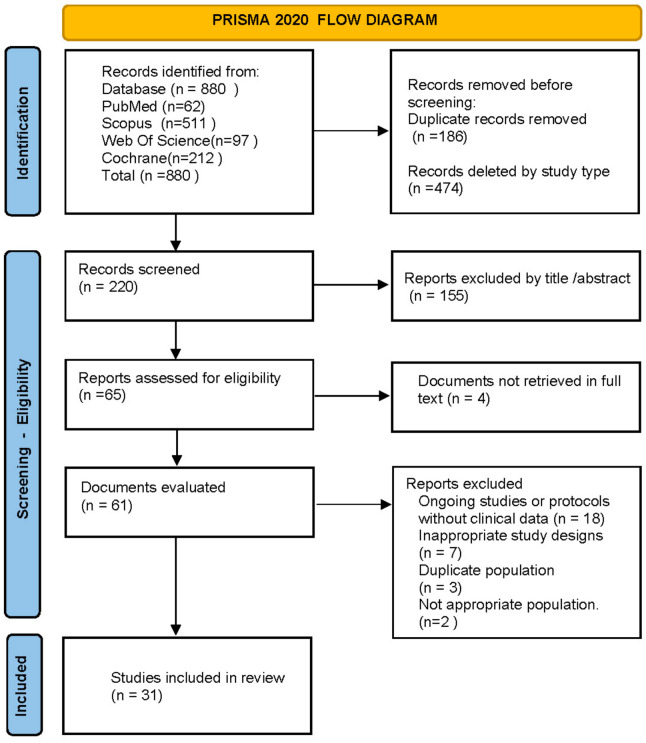
PRISMA flow diagram.

Full-text copies of the 65 potentially relevant articles were sought, with 61 successfully retrieved. A comprehensive full-text review of these documents was conducted to confirm strict alignment with the predefined inclusion and exclusion criteria. Ultimately, 31 publications met all eligibility criteria and were included in the systematic review. Of these, 22 studies focused on light therapy and 9 on dietary nutritional supplementation. Overall, most studies were judged to have either low risk of bias or some concerns according to the Cochrane RoB 2 tool. The main sources of potential bias were related to masking procedures and deviations from intended interventions, whereas outcome assessment was generally considered robust because axial length and refractive outcomes were objectively measured. Figure 1 shows the PRISMA flow diagram describing the detailed study selection process.

### 3.1. General Characteristics of the Literature

#### 3.1.1. Light Therapy Interventions Studies

Regarding light therapy interventions, a total of 22 publications were included. The vast majority (21) were randomized clinical trials (RCTs), with one quasi-experimental study. These studies involved a total of 3068 patients at baseline, with sample sizes ranging from 15 to 336 participants. Of these, approximately 1630 participants were assigned to the intervention groups -which in some cases included multiple study arms with varying doses or intensities of light therapy- and 1438 to the control groups. Table 2 summarizes the comparative characteristics of the 22 included trials.

The intervention duration was 12 months in eleven studies [12,16,17,18,19,20,21,22,23,24,25] and 6 months in eight studies [26,27,28,29,30,31,32,33]. One study presented a follow-up of 3 months [34], another extended to 24 months [35], and the quasi-experimental study [36] had a duration of only 4 weeks. The age of the patients ranged from 6 to 16 years, primarily falling within the school-age period (6 to 12 years). Most participants were children with varying degrees of myopia, predominantly mild to moderate. Notably, specific cohorts with high myopia (>−6.0 D) were included in studies such as those by Liu et al. [18] and Xu et al. [20]. Furthermore, several trials [23,27,31,32,35,37] specifically incorporated non-myopic, pre-myopic, or mildly hyperopic children to evaluate the prophylactic and preventive effect of the intervention on myopia incidence. Geographically, almost all studies were conducted in China, with the exception of a multi-ethnic study by Deen et al. [34] conducted in Australia and a study by Kearney et al. [27] conducted in Scotland. Regarding the methodological quality of the light therapy trials, the Cochrane RoB 2 tool was applied to the 14 included studies. Overall, 1 study (Dong et al. [26]) was judged to have a low risk of bias, while the remaining 13 trials raised some concerns. The primary source of potential bias across this branch was consistently found in Domain 2 (deviations from intended interventions). This widespread outcome was heavily driven by the inherent clinical and practical difficulties of implementing strict masking and double-blind procedures with active laser/optical devices. Conversely, outcome assessment for biometric parameters was considered highly robust because the primary variables—axial length and spherical equivalent—were objectively measured using high-precision automated devices. Detailed domain-specific assessments are maintained in the Supplementary Material (Supplementary File S4). 

**Table 2:** Descriptive comparative table summarizing the general characteristics of the randomized controlled trials included in the Light Therapy Interventions study.

Study	Country	Target Population	Sample Size (IV/CT)	Intervention Protocol	Control	Follow-Up
Cao et al. (2024) [16]	China	High myopia to mild hyperopia (−6.00 D to +3.00 D); 6–12 y.	336 (168/168)	RLRL (650 nm, 0.29 mW) via wearable device. 3 min/session, 2×/day (≥4 h interval), 7 days/wk.	SV spectacles (myopes) or None (non-myopes)	12 mo.
Chen et al. (2023) [22]	China	Myopia (−0.75 D to −6.00 D); 6–13 y.	102 (51/51)	RLRL (635 nm, 0.35 mW). 3 min/session, 2×/day (≥4 h interval), 7 days/wk	SV spectacles	15 mo.
Deen et al. (2024) [34]	Australia	Myopia (−0.50 D to −5.00 D); 8–13 y. (Multiethnic)	34 (16/18)	RLRL (650 nm, 2.0 mW). 3 min/session, 2×/day (>4 h interval), 5 days/wk	SV spectacles	3 mo.
Dong et al. (2023) [26]	China	Myopia (≥−0.50 D); 7–12 y.	112 (56/56)	RLRL (0.29 mW). 3 min/session, 2×/day (≥4 h interval), 7 days/wk. + SV spectacles.	Sham light (0.03 mW) + SV spectacles	6 mo.
He et al. (2023) [23]	China	Pre-myopia (−0.50 D to +0.50 D) with myopic parent; 6–11 y.	278 (139/139)	RLRL (650 nm, 2.0 mW). 3 min/session, 2×/day (≥4 h interval), 5 days/wk.	None	12 mo.
Jiang et al. (2022) [12]	China	Myopia (−1.00 D to −5.00 D); 8–13 y.	264 (119/145)	RLRL (650 nm, 0.29 mW, ~1600 lux). 3 min/session, 2×/day (≥4 h interval), 5 days/wk.	SV spectacles	12 mo.
Kearney et al. (2025) [27]	Scotland	Myopia (up to −6.00 D); 7–13 y.	15 (4/7/4)	IV1: Bright light lamp (10,000 lux). 30 min/day, 7 days/wk. IV2:Bright light lamp + myopia control spectacles	SV spectacles	6 mo.
Liu L. et al. (2024) [17]	China	Myopia (−1.00 D to −6.00 D) & Pre-myopia; 8–14 y.	170 (85/85)	RLRL (650 nm, ~1600 lux). 3 min/session, 2×/day (≥4 h interval), 7 days/wk.	SV spectacles	12 mo.
Liu G. et al. (2025) [18]	China	High myopia (>−6.00 D); 7–12 y.	202	RLRL (650 nm, ~1600 lux). 3 min/session, 2×/day (≥4 h interval), 7 days/wk. + SV spectacles.	SV spectacles	12 mo.
Liu G. et al. (2026) [35]	China	Pre-myopia (−0.50 D to +0.75 D); 8–13 y.	108 (58/50)	RLRL (650 nm). 3 min/session, 2×/day (≥4 h interval), applied for 1 year.	None	24 mo.
Liu Z. et al. (2024) [25]	China	Pre-myopia (−0.50 D to +0.50 D); 7–12 y.	85 (43/42)	RLRL (650 nm, 0.29 mW, ~1600 lux). 3 min/session, 2×/day (≥4 h interval), 7 days/wk.	None	12 mo.
Tian et al. (2022) [28]	China	Myopia (−0.50 D to −6.00 D); 6–12 y.	224 (112/112)	RLRL (650 nm) via wearable device. 3 min/session, 2×/day (≥4 h interval), 7 days/wk. + SV spectacles.	SV spectacles	6 mo.
Tian et al. (2023) [32]	China	Non-myopes; 6–12 y.	112 (56/56)	RLRL (650 nm) via wearable device. 3 min/session, 2×/day (≥4 h interval), 7 days/wk.	None	6 mo.
Wei et al. (2025) [19]	China	Myopia (−0.50 D to −6.00 D); 6–14 y.	200 (100/100)	RLRL (650 nm, 2.0 mW). 3 min/session, 2×/day (≥4 h interval), 5 days/wk.	SV spectacles	12 mo.
Xiong et al. (2024) [29]	China	Myopia (+0.50 D to −6.00 D); 6–14 y.	73 (36/37)	RLRL (650 nm, 0.9 mW). 3 min/session, 2×/day (≥4 h interval), 7 days/wk. + SV spectacles.	SV spectacles	6 mo.
Xu et al. (2024) [20]	China	High myopia; 6–16 y.	192 (97/95)	RLRL (650 nm, 0.29 mW, ~1600 lux). 3 min/session, 2×/day (≥4 h interval), 7 days/wk. + SV spectacles.	SV spectacles	12 mo.
Yang et al. (2025) [30]	China	Myopia (+0.50 D to −6.00 D); 6–14 y.	60 (30/30)	RLRL (650 nm, 2.00 mW). 3 min/session, 2×/day (≥4 h interval).	SV spectacles	6 mo.
Yang et al. (2025) [24]	China	Myopia (−1 D to −1.50 D); 8–10 y.	116 (29/29/29 /29)	(1) Combination therapy: RLRL (650 nm, 0.39 mW) + Distant Image Screen (DIT) 1 h/day. (2) RLRL (3) DIT	SV spectacles	12 mo.
Zhang et al. (2025) [31]	China	Non-myopes; 7–12 y.	86 (43/43)	RLRL (650 nm). 2×/day	SV spectacles	6 mo.
Zhao et al. (2023) [36]	China	Myopia (<−1 D); 6–14 y.	47	RLRL (650 nm). 3 min/session, 2×/day (≥4 h interval). 7 days/wk. + SV spectacles.	SV spectacles	1 mo.
Zhou L. et al. (2023) [21]	China	Myopia (<−0.75 D); 8–12 y.	43 (24/19)	RLRL (650 nm, ~400 lux). 3 min/session, 2×/day (≥4 h interval) + SV spectacles.	SV spectacles	12 mo.
Zhou W. et al. (2024) [33]	China	Myopia; 6–15 y.	200 (50/50/50 /50)	RLRL (650 nm) at three power levels: 0.37 mW, 0.60 mW, 1.20 mW. 3 min/session, 2×/day (≥4 h interval) + SV spectacles.	SV spectacles	6 mo.

IV: Intervention group; CT: Control group; IV1/IV2: Intervention 1/Intervention 2; y.: years; mo.: months; wk.: weeks; h: hours; min: minutes; D: Diopters; RLRL: Repeated Low-Level Red-Light; nm: nanometers; mW: milliwatts; SV: Single-Vision; DIT: Distant Image Screen.

#### 3.1.2. Nutritional Supplementation Studies

Regarding nutritional interventions, a total of nine publications were included comprising six randomized clinical trials (RCTs) and three quasi-experimental studies. These trials involved a total of 935 patients, with sample sizes ranging from 44 to 180 participants. Of these, 497 participants were allocated to the intervention groups. Table 3 summarizes the comparative characteristics of the included nutritional trials. The intervention duration was 12 months in one study [38], 6 months in two studies [39,40], and 2 months in five studies [41,42,43,44,45]. Exceptionally, one study applied the intervention in premature infants from birth to 40 weeks of postmenstrual age [46]. The age of the patients ranged from the neonatal period to 15 years, primarily falling within the school-age demographic (5 to 14 years). Most participants were school-aged children with varying degrees of refractive error, predominantly mild to moderate myopia. Notably, specific subjects with high myopia were included in the study by Omar et al. [38] and in subgroups within the trials by Klopotskaya et al. [45] and Wahyudi et al. [42]. Furthermore, the clinical trial by Li et al. [40] integrated non-myopic children to evaluate the preventive effect of the nutritional interventions. The review was additionally enriched by the inclusion of pediatric populations with special clinical conditions, providing a more comprehensive perspective: this included extremely premature infants [46], children diagnosed with attention deficit hyperactivity disorder (ADHD) [43], and students exhibiting ocular symptomatology derived from digital screen exposure exceeding 4 h daily [41].

### 3.2. Characteristics of Non-Pharmacological Interventions

#### 3.2.1. Light Therapy Interventions

The light therapy interventions described in the twenty-two studies followed a highly standardized approach, predominantly focusing on repeated low-level red-light (RLRL) therapy, with minor variations in power, frequency of use, and administration method. The vast majority of trials used RLRL desktop devices emitting a wavelength of 650 nm at approximately 16,000 lux. However, some exceptions were noted, such as the use of a 635 nm wavelength [22], a 10,000 lux bright-light lamp [27], and an illuminance of 400 lux [21]. Although most studies employed desktop devices, some implemented wearable eye-level integrated systems [16,28,32]. The clinical regimen was remarkably consistent across RLRL trials, typically consisting of 3 min sessions twice daily with a minimum 4 h interval, administered 5 to 7 days per week, with the exception of Kearney et al. [27], who employed bright light 30 min daily sessions. Output power ranged from low levels (0.29 mW) [12,16,20,25,26] to higher levels (up to 2.0 mW) [19,23,24,29,30,34,35,36], with one trial specifically comparing three different power levels [21]. In most myopic populations, light therapy was combined with standard optical correction, although one study innovatively incorporated a distant image screen (DIT) [24]. Conversely, control groups generally received standard care with single-vision spectacles. Notably, the rigorous double-blind design by Dong et al. [26] utilized a sham light device emitting 0.03 mW, while control groups in preventive studies (targeting pre-myopic or non-myopic children) received no active intervention.

#### 3.2.2. Nutritional Interventions

The nutritional interventions described in the nine studies presented a wide variety of approaches, tailored to the prevention or control of myopia progression and visual fatigue in the pediatric population. One group of studies based their interventions on carotenoids and potent antioxidants, such as astaxanthin [41], lutein estIters [40], or crocetin [39]. Another cluster focused on vitamins and plant extracts, including vitamin D administered alone or combined with sunlight exposure [42], multivitamin gummies enriched with minerals and folic acid [44], or vitamin E combined with bilberry extract or with selenium, lutein, and zeaxanthin [45]. A third approach employed lipids and essential fatty acids, either through early parenteral supplementation with arachidonic acid (AA) and docosahexaenoic acid (DHA) in premature infants [46] or through direct dietary modification using Omega-3/PUFA- enriched eggs [43]. Control groups predominantly received inactive placebos designed to mimic the main intervention (e.g., safflower oil capsules, dextrin preparations, or standard eggs) [39,40,41,43,44]. In the remaining trials, control subjects received no sham supplement, maintaining only their standard care, baseline nutrition or no intervention at all.

### 3.3. Efficacy of Interventions

To rigorously evaluate the quantitative results of the review, the meta-analysis was structured into distinct analytical blocks based on the type of intervention and the evaluated variable. First, the efficacy of light therapy on anatomical impact (axial length) and refractive impact (spherical equivalent) was analyzed. Subsequently, the efficacy of nutritional supplementation was evaluated. It should be noted that a meta-analysis of the nutritional impact on the spherical equivalent was not performed because only two studies provided the necessary statistical data.

#### 3.3.1. Efficacy of Light Therapy on Axial Length

**Figure 2:**
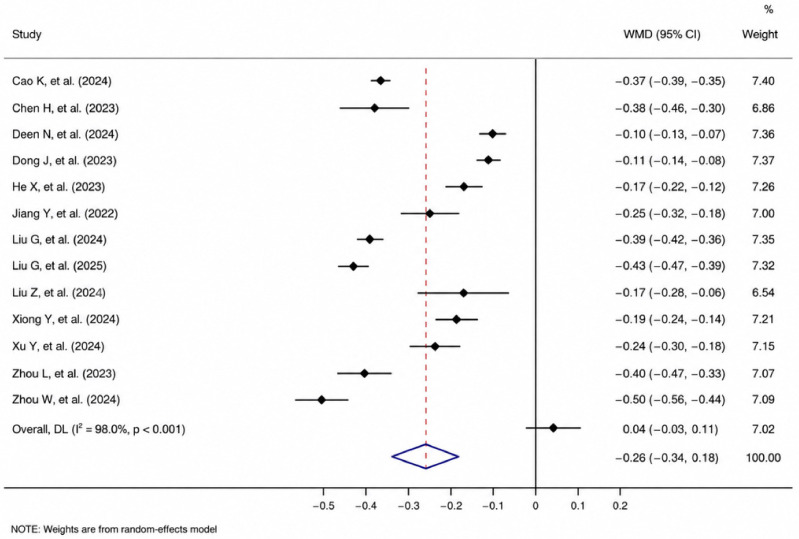
Forest plot of the efficacy of light therapy on axial length. CI, confidence interval; WMD, weighted mean difference. Squares represent the effect estimate for each individual study, with the size of each square proportional to the study weight. Horizontal black lines indicate the 95% confidence intervals. The solid vertical black line represents the line of no effect (WMD = 0). The dashed red vertical line indicates the pooled effect estimate. The blue diamond represents the overall pooled effect estimate, with its width corresponding to the 95% confidence interval. The studies included in this forest plot correspond to References [12,16,18,20,21,22,23,25,26,29,33,34,37].

**Figure 3:**
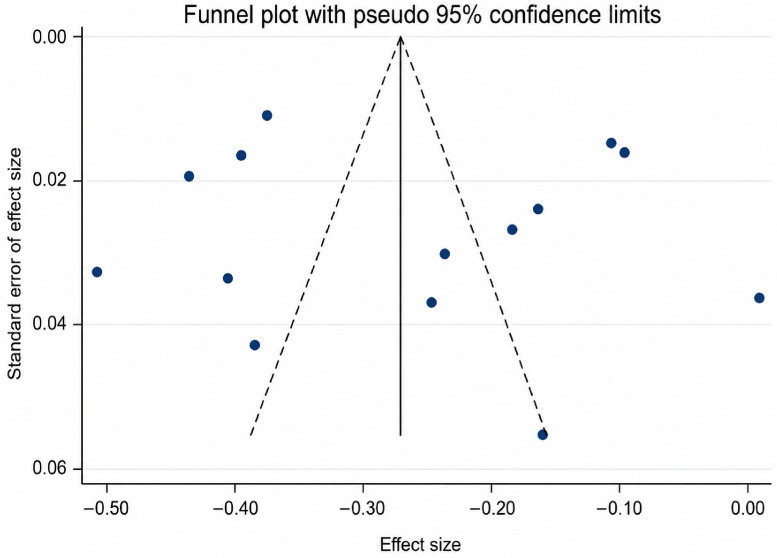
Funnel plot of the axial length results for light therapy. Blue circles represent individual studies. The solid vertical line indicates the pooled effect estimate. The dashed diagonal lines represent the pseudo 95% confidence limits.

A total of fourteen studies met the eligibility criteria for inclusion in the quantitative meta-analysis evaluating the effect of repeated low-level red-light (RLRL) therapy on axial length. Figure 2 displays the forest plot of these results. Of the fourteen included trials, thirteen [12,16,17,18,20,21,22,23,25,27,29,34,35] achieved individual statistical significance, placing their confidence intervals (Cis) in the negative zone, which translates to a clear protective effect in slowing ocular elongation. Only the study by Zhou W. et al. [33] presented a confidence interval crossing the line of no effect (weighted mean difference [WMD] = 0.04; 95% CI −0.03 to 0.11), lacking statistical significance in isolation. Conversely, the trial by Zhou L. et al. [21] reported the greatest reduction in axial length of the entire sample (WMD = −0.50; 95% CI −0.56 to −0.44). In the forest plot, the weights of the studies are highly balanced, varying within a narrow margin from 6.54% to 7.40%. When applying the random-effects model, the pooled mean difference shows an overall reduction of −0.26 mm in axial growth (95% CI −0.34 to −0.18), confirming the overall efficacy of the intervention. However, the heterogeneity index (I^2^) is extremely high (98.0%; *p* < 0.001), underscoring notable variability among the investigations. Additionally, visual inspection of the funnel plot (Figure 3) reveals marked asymmetry and scattering of the points around the overall effect line. A large proportion of the trials fall outside the 95% pseudo-confidence limits, distributing widely both to the left (very drastic axial slowing) and to the right. The presence of points in the lower area of the funnel, corresponding to studies with a larger standard error, indicates that smaller trials with more conservative results were also captured, which does not support the hypothesis of publication bias. Consequently, the scattered morphology corroborates that the high heterogeneity responds to real structural differences between the trials, such as divergences in populations (from pre-myopia to high myopia) and variability in intervention protocols (differences in irradiated power and follow-up times).

#### 3.3.2. Efficacy of Light Therapy on Spherical Equivalent

**Figure 4:**
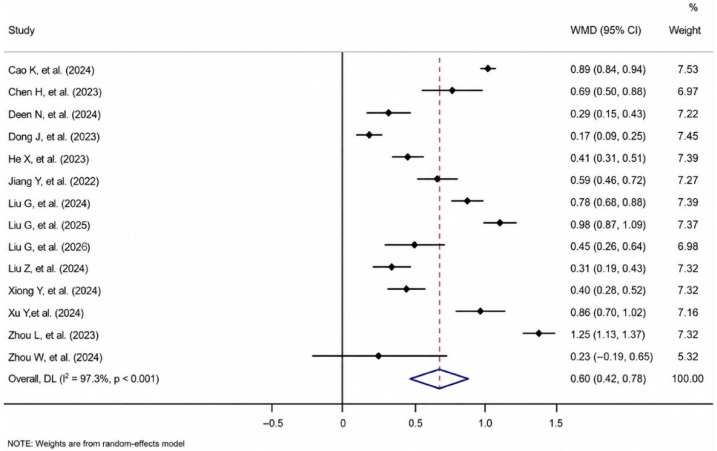
Forest plot of the efficacy of light therapy on the spherical equivalent. CI, confidence interval; WMD, weighted mean difference. Squares represent the effect estimate for each individual study, with the size of each square proportional to the study weight. Horizontal black lines indicate the 95% confidence intervals. The solid vertical black line represents the line of no effect (WMD = 0). The dashed red vertical line indicates the pooled effect estimate. The blue diamond represents the overall pooled effect estimate, with its width corresponding to the 95% confidence interval. The studies included in this forest plot correspond to References [12,16,18,20,21,22,23,25,26,29,33,34,35,37].

**Figure 5:**
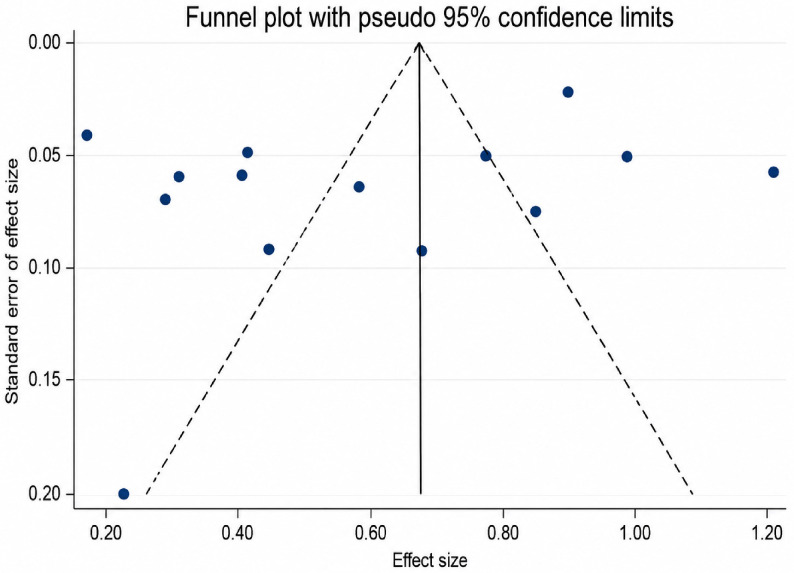
Funnel plot of the spherical equivalent results for light therapy. Blue circles represent individual studies. The solid vertical line indicates the pooled effect estimate. The dashed diagonal lines represent the pseudo 95% confidence limits.

Parallel to the anatomical changes, the refractive impact of RLRL therapy was analyzed by evaluating the spherical equivalent (SE) in the same fourteen clinical trials. It is important to remember that myopic progression is expressed in negative values; therefore, a positive weighted mean difference (WMD) indicates a protective effect. The forest plot (Figure 4) reveals a trend identical to that observed for axial length. Again, thirteen of the fourteen studies show statistically significant results in favour of the treatment. The study by Zhou L. [21] stands out, reporting the greatest protective effect with remarkable refractive preservation (WMD = 1.25 D; 95% CI 1.13 to 1.37). Conversely, the trial by Zhou. W et al. [33] is the sole exception whose confidence interval crosses the no-effect line (WMD = 0.23 D; 95% CI −0.19 to 0.65), lacking individual statistical significance. Using the random-effects model, the overall meta-analysis yields a pooled mean difference of 0.60 diopters (95% CI 0.42 to 0.78), demonstrating a clear clinical benefit of red light in slowing myopia progression. The study weights in the model remain highly balanced, ranging from 5.32% to 7.53%. However, the heterogeneity index is also extremely high (I^2^ = 97.3%; *p* < 0.001). This high heterogeneity is visually corroborated by the wide dispersion observed in the associated funnel plot (Figure 5). The plot shows a markedly asymmetric and scattered distribution of the studies around the overall effect line. Despite this variability, the trials are distributed on both the right side (reporting very pronounced protective effects) and the left side (with more moderate effects). The presence of points in the lower-left quadrant -corresponding to studies with larger standard errors and smaller effect sizes-indicates that investigations with more conservative results were also captured, which inherently discards the hypothesis of a clear publication bias. Although the inclusion of fourteen trials provides adequate visual power to evaluate this plot, a large proportion of the points lie outside the 95% pseudo-confidence limits. This remarkable dispersion is a direct visual reflection of the model’s high heterogeneity. Therefore, the marked asymmetry of the funnel plot does not stem from methodological reporting biases, but from real and profound clinical variations among the trials, such as differences in study designs, administered light powers, and demographic characteristics of the included populations.

#### 3.3.3. Efficacy of Nutritional Supplementation on Axial Length

**Figure 6:**
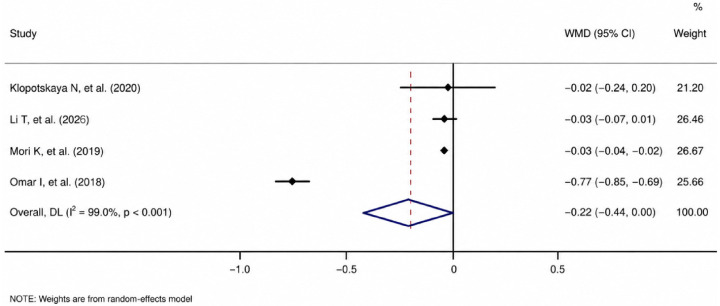
Forest plot of the efficacy of nutritional supplementation on axial length. WMD, weighted mean difference; CI, confidence interval. Squares represent the effect estimate for each individual study, with the size of each square proportional to the study weight. Horizontal black lines indicate the 95% confidence intervals. The solid vertical black line represents the line of no effect (WMD = 0). The dashed red vertical line indicates the pooled effect estimate. The blue diamond represents the overall pooled effect estimate, with its width corresponding to the 95% confidence interval. The studies included in this forest plot correspond to References [38,39,40,45].

**Figure 7:**
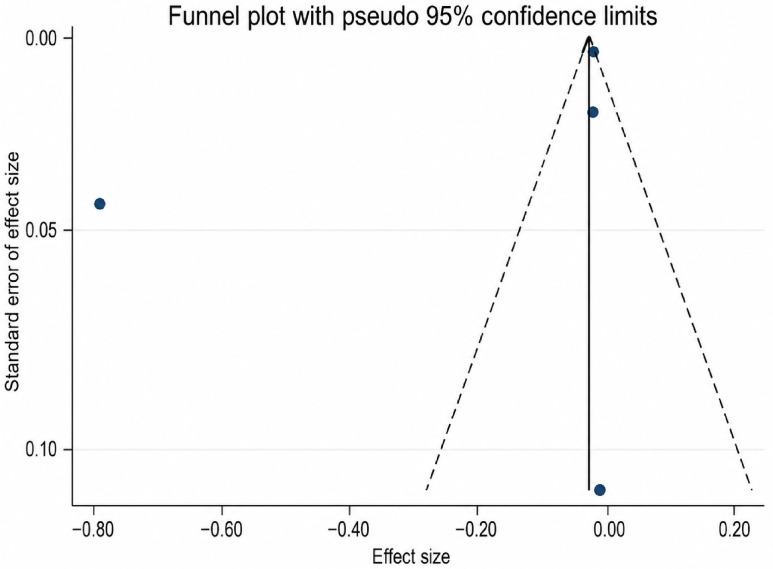
Funnel plot of the axial length results for nutritional supplementation. Blue circles represent individual studies. The solid vertical line indicates the pooled effect estimate. The dashed diagonal lines represent the pseudo 95% confidence limits.

Although a variety of nutritional interventions were identified in the systematic review, only four studies met the eligibility criteria for inclusion in the quantitative meta-analyses of axial length. Figure 6 shows the forest plot of the axial length, and Figure 7 demonstrates the funnel plot of these same results.

Of the studies included, only the works of Omar et al. [38] and Mori et al. [39] reach individual statistical significance, as their confidence intervals (Cis) fall entirely within negative values, demonstrating a protective effect on eye elongation. The study by Omar et al. [38] stands out radically, reporting a highly accentuated reduction compared to its control group (mean difference = −0.77; 95% CI −0.85 to −0.69). Conversely, the studies by Klopotskaya et al. [45] and Li et al. [40] have Cis that cross the null effect line, evidencing the absence of statistically significant findings in those trials in isolation.

In the forest plot, the weights of the studies are highly balanced, ranging evenly from 21.2% to 26.67%. When applying the random-effects model, the pooled mean difference shows an overall reduction of −0.22 mm (95% CI −0.44 to 0.00). This result indicates a protective effect of supplementation; however, it is on the strict borderline of statistical significance. Given that the upper limit of the confidence interval reaches the null value (0.00), it cannot be categorically stated that the effect is independent of chance under conventional criteria (*p* < 0.05). Along with this overall estimate, the heterogeneity index I^2^ is extremely high (99.0%; *p* < 0.001), indicating that the clinical and statistical variability between the studies is extreme.

Additionally, visual inspection of the funnel plot (Figure 7) reveals a clear polarization of the data. Three studies are tightly clustered on the right side with high precision and effect sizes very close to zero. However, a single point far to the left stands out, breaking the symmetry completely. This extreme outlier corresponds to the study by Omar et al. [38]. Its effect size is disproportionately larger than that of the other trials and stands as the primary driver of the massive heterogeneity registered in the overall model.

### 3.4. Other Results: Qualitative and Clinical Outcomes

In addition to the quantitative meta-analyses, a qualitative synthesis of the included studies was performed to evaluate clinically relevant findings that could not be pooled statistically because of methodological and outcome heterogeneity. These additional results are presented below in tabular format and are divided into two major intervention categories: light therapy interventions (Table 4) and nutritional supplementation strategies (Table 5). Key outcomes included clinical efficacy, choroidal and retinal structural changes, functional visual parameters, rebound effects, adherence-related findings, and safety outcomes.

### 3.5. Subgroup Analysis and Sensitivity

Subgroup analysis and sensitivity analysis were not performed in this study due to the small number of studies included in each meta-analysis.

## 4. Discussion

This systematic review and meta-analysis assessed the effectiveness of non-pharmacological and non-optical interventions in pediatric myopia management. The meta-analysis revealed highly significant findings regarding the efficacy of repeated low-level red-light (RLRL, 650 nm) therapy, evidenced by a drastic slowing of ocular elongation (pooled mean difference: −0.26 mm in axial growth) and a notable preservation of the spherical equivalent (0.60 D). In contrast, the efficacy of nutritional interventions showed results bordering the limit of clinical and statistical significance for axial elongation (−0.22 mm), being heavily biased by extreme values in the study by Omar et al. [38].

The findings on light therapy closely align with previous literature. The magnitude of the pooled treatment effect observed for RLRL appears comparable to, and in some studies greater than, that reported for established interventions such as low-dose atropine, orthokeratology, dual-focus contact lenses, or DIMS spectacles. However, these comparisons are indirect and should be interpreted cautiously because of important differences in study design, patient characteristics, outcome definitions, and follow-up duration. To contextualize the true clinical magnitude of our results (a pooled reduction of −0.26 mm in axial growth and a preservation of 0.60 D in a 12-month period), it is imperative to compare the efficacy of RLRL with current standard-of-care treatments. Pivotal clinical trials on low-dose atropine, such as the ATOM2 [47] and LAMP [9] studies, report annual reductions in ocular growth ranging from 0.15 to 0.25 mm depending on the concentration, with 0.05% atropine achieving a 1-year axial brake of 0.21 mm at the expense of a reduction in accommodative amplitude (~1.98 D) and photopic pupil dilation (~1.03 mm). Our meta-analysis demonstrates that RLRL achieves a short-term structural efficacy that equals or even surpasses these pharmacological options, while avoiding such classic anticholinergic side effects.

Similarly, when compared to consolidated non-pharmacological interventions, our data place RLRL a step above in terms of initial biomechanical power. For instance, in the field of peripheral defocus spectacle lenses, the 2-year randomized clinical trial on DIMS technology [48] reported a net axial length braking of 0.34 mm compared to single-vision lenses, while highly aspherical lenses (HAL technology) [49] slowed axial elongation by a mean of 0.35 mm over 2 years, reaching 0.41 mm in children with optimal compliance (>12 h/day). Furthermore, regarding contact lens-based therapies, the landmark ROMIO study [50] on orthokeratology demonstrated a net 2-year axial elongation reduction of 0.27 mm compared to single-vision spectacles. Taken together, these standard optical interventions yield an average control rate that translates to approximately 0.13 to 0.18 mm of axial protection per year. Consequently, the −0.26 mm annual brake found in our RLRL meta-analysis positions red-light therapy as a highly potent tool during the first 12 months of compliance. As supported by recent International Myopia Institute (IMI) [51] reports and the meta-analyses by Youssef et al. [52], RLRL therapy exhibits high initial efficacy, surpassing these consolidated therapies. However, significant questions arise regarding safety. The IMI reports warn that the term “low-level” can be misleading, as laser emissions may approach thermal and photochemical safety limits. In fact, six of the twenty-two studies in our review reported side effects, and parallel literature documents recent cases of photoreceptor damage and vision loss, questioning its long-term safety profile. Recent evidence supports a favourable safety profile for repeated low-level red-light therapy, with retinal adverse events remaining exceedingly uncommon. Nevertheless, continued long-term surveillance remains warranted, particularly because most currently available evidence derives from relatively short-term studies conducted in Asian populations. Since completion of our literature search, additional evidence has become available supporting the favourable safety profile of repeated low-level red-light therapy. A recent systematic review [53] identified only isolated retinal adverse events despite several years of clinical use, while a multicentre three-year real-world study [54] confirmed sustained efficacy without new major safety signals. These findings strengthen the current evidence supporting the clinical use of RLRL, although continued long-term surveillance remains advisable, particularly as most available studies have been conducted in Asian populations.

Regarding the analyses of nutritional supplementation, the lack of primary structural impact is supported by the latest IMI consensus, which does not propose diet as a determining factor for biomechanical myopia control. The IMI clarifies that the protective effect of time outdoors is not mediated by vitamin D synthesis, but by exposure to bright environmental light (>1000 lux), which increases retinal dopamine release, acting as a regulatory “brake” that slows excessive axial elongation [55]. This effect is likely enhanced by a broader spectral composition and reduced peripheral defocus. Nevertheless, and in agreement with a recent adult meta-analysis by Martinez-Perez et al. [56], our qualitative findings suggest that supplementation (e.g., antioxidants, lutein) does provide substantial functional benefits. Its clinical utility lies not in the biomechanical control of the globe, but in its adjuvant role for retinal neuroprotection and the relief of asthenopia in the face of high visual demands.

These results reveal an important clinical paradox: while the quantitative efficacy of red light is indisputable, its practical translation faces significant barriers. The IMI Global Trends document [51] reveals that currently more than 55% of professionals worldwide would not prescribe this intervention due to concerns regarding retinal damage and the high risk of a “rebound effect”, which triggers accelerated myopia progression upon treatment cessation -a finding corroborated by the trials of Chen et al. [22] and Liu et al. [35]. Although rebound after treatment discontinuation has been reported, recent expert recommendations suggest that gradual tapering strategies, rather than abrupt cessation, may substantially reduce this phenomenon [57]. Future clinical trials should further evaluate standardized discontinuation protocols to optimize long-term treatment outcomes. Consequently, our findings support a tiered clinical approach. RLRL should not be prescribed indiscriminately as a first-line or preventive population-wide strategy; instead, it should be reserved for “fast progressors” -whose therapeutic response is significantly higher- under strict informed consent and constant retinal monitoring via OCT.

Among the strengths of this review are the rigorous application of the PRISMA methodology, the exclusive inclusion of RCTs, and the dual evaluation of structural (axial length) and refractive (spherical equivalent) outcomes, granting high robustness to the results. Furthermore, it adds value by assessing both physical (RLRL) and metabolic (nutrition) approaches within the same document. However, several limitations must be considered. The very high statistical heterogeneity observed across pooled analyses represents one of the major limitations of the current evidence. Differences in baseline refractive status, inclusion of both prevention and progression studies, follow-up duration, treatment protocols, irradiance levels, and control groups likely contributed substantially to the observed variability. Consequently, the pooled estimates should be interpreted with caution. An additional source of heterogeneity is the inclusion of both prevention studies in pre-myopic or non-myopic children and progression studies in established myopia. Although both evaluate myopia control, they represent distinct clinical scenarios and may involve partially different biological mechanisms. First, the quantitative models for light therapy exhibited high heterogeneity, reflecting a lack of standardization in treatment protocols. Differences in baseline refractive status, inclusion of both prevention and progression studies, follow-up duration, treatment protocols, and control groups likely contributed substantially to the high heterogeneity observed across studies. Second, the scarcity of nutritional trials with standardized data prevented a quantitative analysis of the spherical equivalent, while the axial length model was distorted by a clear outlier [38]. Another important limitation is that the vast majority of RLRL studies have been conducted in Chinese pediatric populations. Therefore, caution is warranted when extrapolating these findings to children from other geographic regions, where genetic background, environmental exposures, educational systems, and lifestyle factors may differ substantially. There is a marked population and geographic bias: the overwhelming majority of trials originate from Asia. Given that myopia prevalence, ocular anatomy, and scleral biomechanical response differ significantly between ethnicities, direct extrapolation to Caucasian populations must be done with caution. Finally, limited follow-up periods (6 to 24 months) are insufficient to definitively evaluate long-term retinal safety. Although reduction in axial elongation is widely accepted as a surrogate marker of myopia control, evidence demonstrating sustained reductions in myopia-related morbidity after treatment discontinuation remains limited. Long-term prospective studies are therefore required to determine whether these anatomical benefits translate into permanent modification of disease progression.

Considering these findings, future research should prioritize multicentre clinical trials in Caucasian populations with larger sample sizes and mandatory follow-up protocols after device withdrawal to quantify the real “rebound effect”. Future systematic reviews should also evaluate specific clinical populations separately (pre-myopia, incident myopia, progressive myopia and high myopia) and incorporate individual patient characteristics to better explain the heterogeneity currently observed across RLRL studies.

Beyond the evaluation of emerging therapies, our findings reinforce the importance of evidence-based public health strategies for myopia prevention. Repeated low-level red-light (RLRL) therapy appears to be a promising non-pharmacological intervention for reducing axial elongation and slowing myopia progression, although further long-term studies in geographically and ethnically diverse populations are required to confirm its safety and effectiveness. In contrast, current evidence does not support a consistent clinically meaningful effect of nutritional supplementation on myopia progression, highlighting the need for further research to clarify the role of individual nutritional strategies. Until more robust evidence becomes available, preventive measures with well-established efficacy and safety—such as increasing time spent outdoors, optimizing classroom lighting conditions, or promoting healthy visual habits—should remain the cornerstone of pediatric myopia prevention.

## Figures and Tables

**Table 1:** Search strategy based on keywords used in PubMed, Scopus, Web of Science and Cochrane.

Database	Search Terms	Articles Found
PubMed	(“Myopia”[Mesh] OR “Myopia, Degenerative”[Mesh] OR “Refractive Errors”[Mesh] OR “Myopi*”[Title/Abstract] OR “Refractive error*”[Title/Abstract] OR “Short-sight*”[Title/Abstract] OR “Nearsightedness*” [Title/Abstract] OR “Near-sight*”[Title/Abstract] OR “Axial length”[Title/Abstract] OR “axial elongation”[Title/Abstract] OR “Refraction”[Title/Abstract]) AND (“diet”[MeSH Terms] OR “Vitamins”[Mesh] OR “Dietary Supplements”[Mesh] OR “Carotenoids”[Mesh] OR “Ascorbic Acid”[Mesh] OR “Vitamin D”[Mesh] OR “Vitamin E”[Mesh] OR “Vitamin K”[Mesh] OR “Micronutrients”[Mesh] OR “vaccinium myrtillus”[MeSH] OR “zinc”[MeSH] OR “calcium”[MeSH] OR “magnesium”[MeSH] OR “selenium”[MeSH] OR “copper”[MeSH] OR “iron”[MeSH] OR “herbal medicine”[MeSH] OR “phytotherapy”[MeSH] OR “Phototherapy”[MeSH] OR “Dietary Proteins”[MeSH] OR “diet”[Title/Abstract] OR “Vitamin*”[Title/Abstract] OR “Dietary Supplement*”[Title/Abstract] OR “antioxidant*”[Title/Abstract] OR “Micronutrient*”[Title/Abstract] OR “Carotenoid*”[Title/Abstract] OR “Vitamin D”[Title/Abstract] OR “Vitamin E”[Title/Abstract] OR “Multivitamin*”[Title/Abstract] OR “Vitamin K”[Title/Abstract] OR “Retinol”[Title/Abstract] OR “Thiamin*”[Title/Abstract] OR “Riboflavin”[Title/Abstract] OR “Niacin”[Title/Abstract] OR “Pyridoxin*”[Title/Abstract] OR “Vitamin C”[Title/Abstract] OR “Biotin”[Title/Abstract] OR “Folic Acid”[Title/Abstract] OR “Folate”[Title/Abstract] OR “Cyanocobalamin”[Title/Abstract] OR “Vitamin B12”[Title/Abstract] OR “Cobalamin”[Title/Abstract] OR “Ascorbic Acid”[Title/Abstract] OR “Tocopherol*”[Title/Abstract] OR “Cholecalciferol”[Title/Abstract] OR “Ergocalciferol”[Title/Abstract] OR “25-hydroxyvitamin D”[Title/Abstract] OR “25(OH)D”[Title/Abstract] OR “Calcitriol”[Title/Abstract] OR “lutein*”[Title/Abstract] OR “zeaxanthin*”[Title/Abstract] OR “anthocyanin*”[Title/Abstract] OR “anthocyanoside*”[Title/Abstract] OR “vaccinium myrtillus extract”[Title/Abstract] OR “bilberry”[Title/Abstract] OR “vaccinium”[Title/Abstract] OR “vaccinium myrtillus”[Title/Abstract] OR “bilberries”[Title/Abstract] OR “Lycium barbarum”[Title/Abstract] OR “goji”[Title/Abstract] OR “nutraceutical*”[Title/Abstract] OR “functional food*”[Title/Abstract] OR “omega 3 fatty acid*”[Title/Abstract] OR “omega 6 fatty acid*”[Title/Abstract] OR “fatty acid*”[Title/Abstract] OR “zinc*”[Title/Abstract] OR “calcium*”[Title/Abstract] OR “magnesium*”[Title/Abstract] OR “selenium*”[Title/Abstract] OR “copper*”[Title/Abstract] OR “iron”[Title/Abstract] OR “Dietary Protein*”[Title/Abstract] OR “Protein intake”[Title/Abstract] OR “Red light”[Title/Abstract] OR “RLRL”[Title/Abstract] OR “Low-level light”[Title/Abstract] OR “Low level light”[Title/Abstract] OR “Photobiomodulation”[Title/Abstract] OR “Light therapy”[Title/Abstract] OR “Phototherapy”[Title/Abstract] OR “Red Light Phototherap*”[Title/Abstract]) AND (“Child”[Mesh] OR “Adolescent”[Mesh] OR “Infant”[Mesh] OR Child*[Title/Abstract] OR Adolesc*[Title/Abstract] OR Teen*[Title/Abstract] OR Youth*[Title/Abstract] OR Student*[Title/Abstract] OR Pediatric*[Title/Abstract] OR Paediatric*[Title/Abstract] OR “School child*”[Title/Abstract])	62
Cochrane	([mh “Myopia”] OR [mh “Myopia, Degenerative”] OR myop*:ti,ab,kw OR (refractive NEXT error*):ti,ab,kw OR (short NEXT sight*):ti,ab,kw OR (near NEXT sight*):ti,ab,kw OR (axial NEXT length):ti,ab,kw OR refraction:ti,ab,kw OR myopi*:ti,ab,kw OR (axial NEXT elongation):ti,ab,kw) AND ([mh “Diet”] OR [mh “Vitamins”] OR [mh “Dietary Supplements”] OR [mh “Carotenoids”] OR [mh “Ascorbic Acid”] OR [mh “Vitamin D”] OR [mh “Vitamin E”] OR [mh “Vitamin K”] OR [mh “Micronutrients”] OR [mh “Vaccinium myrtillus”] OR [mh “Zinc”] OR [mh “Calcium”] OR [mh “Magnesium”] OR [mh “Selenium”] OR [mh “Copper”] OR [mh “Iron”] OR [mh “Herbal Medicine”] OR [mh “Phytotherapy”] OR [mh “Phototherapy”] OR [mh “Dietary Protein”] OR diet:ti,ab,kw OR Vitamin*:ti,ab,kw OR “Dietary Supplement*”:ti,ab,kw OR antioxidant*:ti,ab,kw OR Micronutrient*:ti,ab,kw OR Carotenoid*:ti,ab,kw OR “Vitamin D”:ti,ab,kw OR “Vitamin E”:ti,ab,kw OR Multivitamin*:ti,ab,kw OR “Vitamin K”:ti,ab,kw OR Retinol:ti,ab,kw OR Thiamin*:ti,ab,kw OR Riboflavin:ti,ab,kw OR Niacin:ti,ab,kw OR Pyridoxin*:ti,ab,kw OR “Vitamin C”:ti,ab,kw OR Biotin:ti,ab,kw OR “Folic Acid”:ti,ab,kw OR Folate:ti,ab,kw OR Cyanocobalamin:ti,ab,kw OR “Vitamin B12”:ti,ab,kw OR Cobalamin:ti,ab,kw OR “Ascorbic Acid”:ti,ab,kw OR Tocopherol*:ti,ab,kw OR Cholecalciferol:ti,ab,kw OR Ergocalciferol:ti,ab,kw OR “25-hydroxyvitamin D”:ti,ab,kw OR “25(OH)D”:ti,ab,kw OR Calcitriol:ti,ab,kw OR lutein*:ti,ab,kw OR zeaxanthin*:ti,ab,kw OR anthocyanin*:ti,ab,kw OR anthocyanoside*:ti,ab,kw OR “vaccinium myrtillus extract”:ti,ab,kw OR bilberry:ti,ab,kw OR vaccinium:ti,ab,kw OR “vaccinium myrtillus”:ti,ab,kw OR bilberries:ti,ab,kw OR “Lycium barbarum”:ti,ab,kw OR goji:ti,ab,kw OR nutraceutical*:ti,ab,kw OR “functional food*”:ti,ab,kw OR “omega 3 fatty acid*”:ti,ab,kw OR “omega 6 fatty acid*”:ti,ab,kw OR “fatty acid*”:ti,ab,kw OR zinc*:ti,ab,kw OR calcium*:ti,ab,kw OR magnesium*:ti,ab,kw OR selenium*:ti,ab,kw OR copper*:ti,ab,kw OR iron:ti,ab,kw OR “Dietary protein”:ti,ab,kw OR “Protein intake”:ti,ab,kw OR “Red light”:ti,ab,kw OR RLRL:ti,ab,kw OR “Low-level light”:ti,ab,kw OR “Low level light”:ti,ab,kw OR Photobiomodulation:ti,ab,kw OR “Light therapy”:ti,ab,kw OR Phototherapy:ti,ab,kw OR “Red Light Phototherap*”:ti,ab,kw) AND ([mh “Child”] OR [mh “Adolescent”] OR child*:ti,ab,kw OR adolesc*:ti,ab,kw OR teen*:ti,ab,kw OR pediatric*:ti,ab,kw OR paediatric*:ti,ab,kw OR (school NEXT child*):ti,ab,kw OR student*:ti,ab,kw)	212
Web of Science	TS = (Myopia OR “Degenerative myopia” OR “Refractive error*” OR “Short-sight*” OR “Near-sight*” OR “Axial length” OR Refraction OR myopi* OR “axial elongation”) AND TS = (Vitamin* OR Multivitamin* OR “Dietary Supplement*” OR Nutrition* OR Diet* OR Nutraceutical* OR “Functional food*” OR Micronutrient* OR Antioxidant* OR Retinol OR “Vitamin A” OR “Vitamin B Complex” OR Thiamin* OR Riboflavin OR Niacin OR Pyridoxin* OR Biotin OR Folate OR “Folic Acid” OR Cobalamin OR Cyanocobalamin OR “Vitamin B12” OR “Ascorbic Acid” OR “Vitamin C” OR Tocopherol* OR “Vitamin D” OR “Vitamin E” OR “Vitamin K” OR Cholecalciferol OR Ergocalciferol OR “25(OH)D” OR “25-hydroxyvitamin D” OR Calcitriol OR “Trace Elements” OR Minerals OR Calcium OR Magnesium OR Zinc OR Iron OR Copper OR Selenium OR “Fatty Acid*” OR “Omega 3” OR “Omega 6” OR DHA OR “Fish oil” OR Carotenoid* OR Lutein* OR Zeaxanthin* OR Anthocyanin* OR Anthocyanoside* OR “Vaccinium myrtillus” OR Bilberry OR Bilberries OR Vaccinium OR “Lycium barbarum” OR Goji OR “Herbal Medicine” OR Phytotherapy OR “Dietary protein*” OR “Protein intake”OR “Red light” OR “RLRL” OR “Low-level light” OR “Low level light” OR “Photobiomodulation” OR “Light therapy” OR “Phototherapy” OR “Red Light Phototherap*” OR Phototherap*) AND TS = (Child* OR Adolescent* OR Infant* OR Teen* OR Youth* OR Student* OR Pediatric* OR Paediatric* OR “School child*”)	97
Scopus	TITLE-ABS (Myopia OR “Degenerative myopia” OR “Refractive error*” OR “Short-sight*” OR “Near-sight*” OR “Axial length” OR Refraction OR myopi* OR “axial elongation”) AND TITLE-ABS (Vitamin* OR Multivitamin* OR “Dietary Supplement*” OR Nutrition* OR Diet* OR Nutraceutical* OR “Functional food*” OR Micronutrient* OR Antioxidant* OR Retinol OR “Vitamin A” OR “Vitamin B Complex” OR Thiamin* OR Riboflavin OR Niacin OR Pyridoxin* OR Biotin OR Folate OR “Folic Acid” OR Cobalamin OR Cyanocobalamin OR “Vitamin B12” OR “Ascorbic Acid” OR “Vitamin C” OR Tocopherol* OR “Vitamin D” OR “Vitamin E” OR “Vitamin K” OR Cholecalciferol OR Ergocalciferol OR “25(OH)D” OR “25-hydroxyvitamin D” OR Calcitriol OR “Trace Elements” OR Minerals OR Calcium OR Magnesium OR Zinc OR Iron OR Copper OR Selenium OR “Fatty Acid*” OR “Omega 3” OR “Omega 6” OR DHA OR “Fish oil” OR Carotenoid* OR Lutein* OR Zeaxanthin* OR Anthocyanin* OR Anthocyanoside* OR “Vaccinium myrtillus” OR Bilberry OR Bilberries OR Vaccinium OR “Lycium barbarum” OR Goji OR “Herbal Medicine” OR Phytotherapy OR “Dietary protein*” OR “Protein intake” OR “Red light” OR “RLRL” OR “Low-level light” OR “Low level light” OR “Photobiomodulation” OR “Light therapy” OR “Phototherapy” OR “Red Light Phototherap*” OR Phototherap*) AND TITLE-ABS (Child* OR Adolescent* OR Infant* OR Teen* OR Youth* OR Student* OR Pediatric* OR Paediatric* OR “School child*”)	511

**Table 3:** Descriptive comparative table summarizing the general characteristics of the randomized controlled trials included in the Nutritional Supplementation study.

Study	Country	Target Population	Sample Size (IV/CT)	Intervention Protocol	Control	Follow-Up
Hecht et al. (2025) [41]	India	Children with digital visual fatigue (≥4 h/day) and computer vision syndrome; 10–14 y.	64 (32/32)	Astaxanthin 4 mg/day (AstaReal^®^, AB, Nacka, Sweden). Applied for 84 days.	Placebo	84 days (~3 mo.)
Klopotskaya et al. (2020) [45]	Ukraine	Mild, moderate, and high myopia; 3 to >12 y.	44 (34/10)	Baseline treatment + Oral antioxidant (Pro-Visio: selenium 50 mcg, Vit E 30 mg, lutein, zeaxanthin, bilberry extract 20 mg). 1 dose/day (>12 y) or 0.5 dose/day (3–12 y) for 3 months.	Baseline treatment + B-group vitamins	6 mo.
Li et al. (2025) [40]	China	Myopic and non-myopic children; 8–12 y.	180 (90/90)	Oral supplementation with lutein ester, dextrin, and maltodextrin (8 mg). 1 dose/day for 6 months.	Placebo (dextrin and maltodextrin)	6 mo.
Lundgren et al. (2023) [46]	Sweden	Extremely premature infants (<28 weeks gestation).	178 (84/94)	Enteral supplementation of PUFAs (Arachidonic acid and DHA), initiated within 72 h of birth until 40 weeks postmenstrual age.	Standard nutrition	2.5 years (corrected age)
Mori et al. (2019) [39]	Japan	Myopia (−1.50 D to −4.50 D); 6–12 y.	69 (39/30)	Oral supplementation with 7.5 mg crocetin (carotenoid). 1 dose/day for 24 weeks.	Placebo (safflower oil capsule)	24 weeks (~6 mo.)
Omar et al. (2018) [38]	Egypt	High myopia (>−6.00 D); 6–14 y.	64 (32/32)	Oral supplementation with Difrarel E 50 mg (bilberry extract + vitamin E). 2 tablets/day, 20 days/month for 1 year.	No supplements	12 mo. (up to 24 mo.)
Qader et al. (2019) [44]	Iraq	Primary school children (4th–6th grade) with refractive errors.	74 (37/37)	“Happy Gummy” multivitamin (calcium, iron, zinc, folic acid, fruit juice). 1 tablet/day for 3 months.	Placebo	3 mo.
Wahyudi et al. (2020) [42]	Indonesia	Moderate to high myopia (−2.00 D to −6.00 D); 12–15 y.	83 (20/20/20/23)	Three intervention arms: (X1) Vitamin D 400 IU/day; (X2) Sunlight exposure 20 min/day (3×/week); (X3) Combined Vit D (400 IU) + Sunlight. Applied for 12 weeks.	No treatment	12 weeks (~3 mo.)
Wu et al. (2015) [43]	China	Children with ADHD or lower IQ; 7–12 y.	179 (89/90)	Dietary modification: PUFA- and omega-3 (EPA and DHA) enriched egg. 1 egg/day for 3 months.	Standard ordinary egg	3 mo.

IV: Intervention group; CT: Control group; y.: years; mo.: months; h: hours; min: minutes; D: Diopters; mg: milligrams; mcg: micrograms; Vit: Vitamin; PUFA: Polyunsaturated Fatty Acids; DHA: Docosahexaenoic Acid; EPA: Eicosapentaenoic Acid; IU: International Units; ADHD: Attention Deficit Hyperactivity Disorder; IQ: Intelligence Quotient.

**Table 4:** Additional Qualitative and Clinical Outcomes of Light Therapy Interventions.

Study	Key Qualitative/Clinical Outcomes
Chen et al. [22]	Improved accommodative function at 12 months. However, a rebound effect was noted 3 months after cessation, characterized by accelerated myopia progression (AL: +0.16 mm; SE: −0.20 D) and loss of subfoveal choroidal thickness.
He et al. [23]	Reduced the relative incidence of myopia by 33.4% at 12 months (up to 54.1% with strict adherence). Highly effective in pre-myopes with hyperopic reserve, but minimal efficacy in borderline subjects. Protective effect on uncorrected visual acuity (UCVA). Long-term safety confirmed; mild adverse events limited to prolonged afterimages.
Kearney et al. [27]	Morning bright-light therapy was feasible and well-tolerated (only one case of asthenopia). Showed a favourable clinical trend toward reduced elongation and choroidal thickening, though without statistical significance due to the small sample size (*n* = 15)
Liu et al. [25]	Significantly greater anatomical efficacy in subjects with established myopia vs. pre-myopes (axial containment of −0.112 mm). Nevertheless, in pre-myopes, it maintained notable prophylactic efficacy, reducing new myopia incidence to 2.5% (vs. 19.4% in control).
Liu et al. [35]	Significantly reduced incidence in the first year, but lost significance at 24 months as subjects approached the myopic threshold. A marked rebound effect was observed after abrupt suspension, with accelerated axial elongation (+0.08 mm vs. control) and drastic choroidal thinning.
Liu et al. [18]	In high myopia, induced significant and sustained thickening of the choroid and para/perifoveal retina at 12 months. Early choroidal thickening (first month) demonstrated high predictive value for 12-month axial control efficacy. Optimal safety profile with no fundus toxicity.
Tian et al. [28]	Significantly slowed myopia progression at 6 months. Axial length shortened in 63.74% of children, and 51.65% showed refractive improvement. Increased choroidal thickness observed. No adverse effects reported.
Tian et al. [32]	Significantly reduced the risk of myopia onset at 6 months (1.8% incidence vs. 12.5% in control). Accompanied by reduced axial elongation and a significant hyperopic shift.
Wei et al. [19]	Induced marked and sustained choroidal thickening, contrasting with thinning in the control group. Improvements in blood flow were transient (peaking at 3 months). Increased choroidal thickness at 6 months was an excellent early predictor of long-term myopia control.
Yang et al. [30]	At 6 months, treated patients showed no statistically significant variations in axial length or spherical equivalent from baseline. The demographic analysis revealed higher efficacy in younger children (6–8 years; 93.3% success) and females (82.35% vs. 44.4% control).”
Yang et al. [24]	The combination therapy (RLRL + Distant Image Screen) showed a synergistic effect: axial length grew by only 0.04 mm (vs. 0.42 mm in control), with remarkable choroidal thickening (+15 µm). A hyperopic shift was observed in 79.3% of the combination group.
Zhang et al. [31]	Notable protective efficacy at 6 months for axial elongation and refractive progression. Significant choroidal thickening (+30 µm). Transient, asymptomatic hyperreflectivity in the retinal pigment epithelium was observed in 7% of cases, resolving fully 0.5–3 months post-suspension
Zhao et al. [36]	At 4 weeks, significant subfoveal choroidal thickening was observed. Therapy improved choroidal blood perfusion cumulatively over time, without causing toxic damage or abnormal changes in vascular density.

**Table 5:** Additional Qualitative and Clinical Outcomes of Nutritional Supplementation.

Study	Key Qualitative/Clinical Outcomes
Lundgren et al. [46]	No statistically significant effect of supplementation on visual acuity was detected at 2.5 years. Hyperopia (16.8%) was more common than myopia (4%), with myopia found exclusively in the control group.
Mori et al. [39]	At 24 weeks, crocetin demonstrated a protective structural effect by inducing significant choroidal thickening, in contrast to the progressive thinning observed in the placebo group.
Omar et al. [38]	High prophylactic efficacy during the first year of intervention (69% containment in refractive progression and 74% in axial elongation compared to the control). Treatment suspension in the second year did not induce a rebound effect, achieving biometric and refractive stabilization that lasted for 24 months.
Qader et al. [44]	In children with visual fatigue, an “excellent” improvement in visual acuity was observed in the intervention group. A strong, statistically significant association was found between the improvement of refractive errors and multivitamin intake compared to the placebo.
Wahyudi et al. [42]	There was a decrease in the degree of myopia across all intervention groups. A strong negative association was demonstrated: a greater increase in serum vitamin D and sun exposure resulted in greater inhibition of myopia progression, with the combination therapy showing the most significant differences.
Wu et al. [43]	Children in both groups experienced a significant improvement in their vision, but the improvement in visual acuity in the omega-3 supplemented group was significantly superior.

## Data Availability

The original contributions presented in the study are included in the article/Supplementary Material; further inquiries can be directed to the corresponding author.

## Supplementary Materials

The following supporting information can be downloaded at: https://www.mdpi.com/article/10.3390/children13070915/s1, Supplementary File S1: PRISMA Checklist; Supplementary File S2: PROSPERO Protocol (Registration No. CRD420261301596); Supplementary File S3: Completed AMSTAR-2 Critical Appraisal Checklist [58]; Supplementary File S4: Cochrane Risk of Bias 2 Assessment.
