# Supplementary material for: Non-Pharmacological and Non-Optical Interventions for Myopia Prevention and Control in Children: A Systematic Review and Meta-Analysis
Source: Children (Basel). 2026 Jul 10;13(7):915. doi: 10.3390/children13070915 (PMC13406435; doi:10.3390/children13070915)
Supplement: Supplementary file 1 [file children-13-00915-s001.zip › Supplementary File S2. PROSPERO.pdf]

Impact of non-pharmacological interventions on myopia incidence and progression in children: a systematic review and meta-analysis

Claudia Domínguez Morras, Laura Moreno, Miren Paniagua, Isabel Castro-Garrido, Alejandro Fernández-Montero

PREVIEW

Citation

Claudia Domínguez Morras, Laura Moreno, Miren Paniagua, Isabel Castro-Garrido, Alejandro Fernández-Montero. Impact of non-pharmacological interventions on myopia incidence and progression in children: a systematic review and meta-analysis. Not yet published.

REVIEW TITLE AND BASIC DETAILS

Review title

Impact of non-pharmacological interventions on myopia incidence and progression in children: a systematic review and meta-analysis

Condition or domain being studied

Myopia; Non-Pharmacological Interventions; Usual Care; Placebo; Paediatric Care

Children and adolescents (aged < 18 years). Participants may be non-myopic (for incidence analysis) or myopic (for progression analysis)

Non-pharmacological interventions aiming to control myopia onset or progression, specifically:

- Light therapies: specifically Repeated Low-Level Red-Light (RLRL) therapy, Low-Level Light Therapy (LLLT), or photobiomodulation delivered via desktop or wearable devices.
- Nutritional interventions: including dietary supplements (e.g., lutein, omega-3 fatty acids, anthocyanins), vitamins, minerals, or specific dietary modifications.

Only non- medical or non-pharmacological interventions will be included; studies evaluating pharmacological treatments, surgical procedures, topical drugs, or optical correction strategies such as contact lenses or other corrective lenses will be excluded. Interventions based solely on behavioral modifications (e.g., increased outdoor time without a specific device) or optical interventions (e.g., Ortho-K, defocus lenses) will be also excluded.

Control groups receiving:

- Placebo (for nutritional studies).
- Sham treatment (for light therapy studies).
- No intervention / Usual care.
- Standard optical correction (single-vision spectacle lenses or contact lenses) without myopia control features.

Main outcomes:

- Change in Spherical Equivalent Refraction (SER) measured in diopters.
- Change in Axial Length (AL) measured in millimeters.
- Myopia incidence or progression based on self-reported diagnosis, defined as participant-reported new onset or worsening of myopia in studies where objective refractive measurements are not available

Rationale for the review

Myopia has emerged as a significant global public health concern, with increasing prevalence among children and adolescents. High myopia is associated with sight-threatening pathologies later in life. While current control strategies such as atropine eye drops and orthokeratology are effective, they may be associated with side effects (e.g., photophobia, blurred near vision) or high costs.

Consequently, there is growing interest in non-pharmacological and non-invasive alternatives But the evidence remains mixed and often limited to observational data. Specifically, Youssef et al. (2024), in a meta-analysis of RCTs, identified

650 nm RLRL as a clinically significant alternative with a favorable safety profile and no structural damage over short periods. However, critical gaps remain regarding its long-term efficacy and the potential rebound effect after suspension.

On the other hand, a recent systematic review by Xu et al. (2025) identified potential associations between specific dietary components and myopia in adolescents. Besides, although previous meta-analyses (Tang et al., 2019) have suggested a general inverse association between serum vitamin D levels and myopia in adults, the evidence in the pediatric population remains controversial. In fact, this association was not statistically significant in the subgroup analysis of children under 18 years of age.

This systematic review aims to synthesize the latest evidence on the efficacy and safety of these specific non-pharmacological interventions (light therapy and nutrition) to provide clear guidance for clinical practice and identify gaps in current research.

Review objectives

What is the efficacy of non-pharmacological interventions(light therapy and nutritional supplementation) in reducing the incidence and progression of myopia in children and adolescents compared to standard care or no intervention?

1. To evaluate the effect of these interventions on the change in Spherical Equivalent Refraction (SER) and Axial Length (AL).
2. To compare the efficacy of these interventions against placebo, sham treatment, or single-vision spectacle lenses.

Keywords

Myopia; Myopia control; Children; Dietary supplements; Light therapy; Repeated low-level red light RLRL

Country

Spain

ELIGIBILITY CRITERIA

Population

Included

Children and adolescents (aged under or equal 18 years). Participants may be non-myopic (for incidence analysis) or myopic (for progression analysis).

Excluded

1. Ocular pathologies other than refractive error (e.g., amblyopia, strabismus, cataract, retinopathy, or active ocular inflammation).
2. Systemic syndromes or diseases affecting ocular growth (e.g., Marfan syndrome, Down syndrome, Stickler syndrome).
3. Concomitant use of atropine or other myopia control interventions during the study period.
4. Studies conducted exclusively in adults or participants older than 18 years old.

Studies reporting data on mixed populations (e.g., including participants >18 years) will only be included if data for the pediatric subgroup (age <18 years) are reported separately or can be extracted. If separate data for the pediatric subgroup are not reported and cannot be extracted from the published article, the study will be excluded to ensure the validity of the findings, given that myopia progression mechanisms and rates differ significantly between growing children and stable adults.

Intervention(s) or exposure(s)

Included

Light Therapy; Low Level Laser Therapy; Non-Pharmacological Interventions; Nutritional supplement; Vitamins

Studies evaluating the following non-pharmacological interventions will be included:

1. Light therapy: Interventions involving the use of Repeated Low-Level Red-Light (RLRL) therapy instruments, Low-Level Light Therapy (LLLT), or photobiomodulation devices

2. Nutritional interventions: Interventions involving oral administration of dietary supplements (lutein, zeaxanthin, omega-3 fatty acids, anthocyanins, vitamins, minerals) of any dose.

Excluded

1. Behavioral interventions relying solely on lifestyle modifications (e.g., increased time outdoors) without the use of specific therapeutic devices
2. Optical interventions for myopia control(orthokeratology, multifocal contact lenses, peripheral defocus spectacle lenses, or bifocal/progressive addition lenses).
3. Pharmacological interventions (atropine, pirenzepine, or 7-methylxanthine).
4. Surgical interventions
5. Combined interventions where the independent effect of the light therapy or nutritional supplement cannot be isolated

Comparator(s) or control(s)

Included
PICO tags selected: Placebo; Sham Intervention ; Usual Care; Contact Lenses

1. Placebo
2. Sham intervention
3. No intervention or usual care
4. Standard optical correction: single-vision spectacle lenses or single-vision contact lenses that do not incorporate myopia control features.

Study design

Both randomized and nonrandomized study types will be included.

Included
We will include Randomized Controlled Trials (RCTs) and Controlled Clinical Trials (CCTs) (quasi-randomized trials) aimed at evaluating the efficacy and safety of the interventions.

Rationale for including CCTs: While RCTs are the gold standard, quasi-randomized trials will be included to maximize the available data, particularly for nutritional interventions where strict RCTs may be less frequent.

Excluded
1. Observational studies (e.g., cohort studies, case-control studies, cross-sectional studies) without an intervention group.

2. Case reports, case series, reviews, editorials, commentaries, and letters to the editor.

3. Animal studies or ex-vivo studies.

4. Conference abstracts where the full text is not available or insufficient data is provided for analysis.

5.Meta-analysis

Context

There will be no geographical restrictions; studies from any country worldwide will be eligible for inclusion. Regarding the setting, the review will include studies conducted in community settings (e.g., schools, home-based interventions) as well as clinical settings (e.g., ophthalmology clinics or hospitals), provided the participants represent the general pediatric population.

SIMILAR REVIEWS

Check for similar records already in PROSPERO

PROSPERO identified a number of existing PROSPERO records that were similar to this one (last check made on 7 February 2026). These are shown below along with the reasons given by that the review team for the reviews being different and/or proceeding.

- Efficacy of Light Therapy for Myopia Prevention in School-Aged Children: A Systematic Review of Randomized Controlled Trials [published 8 May 2025] [CRD420251048146]. The review was judged **not to be similar**
- Updated Meta-Analysis on the Efficacy of Peripheral Defocus Modifying Spectacle Lenses in Controlling Myopia Progression in Children and adolescents. [published 13 January 2026] [CRD420261284162]. The review was judged **not to be similar**

- Efficacy of repeated low-level red light for myopia control in children and adolescents: A systematic review and Meta-Analysis [published 26 March 2025] [CRD420251018947]. The review was judged **not to be similar**

TIMELINE OF THE REVIEW

Date of first submission to PROSPERO
This record has not been submitted.

Review timeline
Start date: 5 February 2026. End date: 16 May 2026.

Date of registration in PROSPERO
This record has not been published.

AVAILABILITY OF FULL PROTOCOL

Availability of full protocol
A full protocol has been written and uploaded to PROSPERO. The protocol will be made available after the review is completed.

SEARCHING AND SCREENING

Search for unpublished studies
Only published studies will be sought.

Main bibliographic databases that will be searched
The main databases to be searched are *CENTRAL - Cochrane Central Register of Controlled Trials*, *PubMed* and *Scopus*.

Other important or specialist databases that will be searched
Web of science

Search language restrictions
There are no language restrictions.

Search date restrictions
Databases will be searched for articles published from 1 January 2000, there are no search end date restrictions.

Other methods of identifying studies
No other methods will be used.

Link to search strategy
A full search strategy has been uploaded to PROSPERO. The PDF may be accessed through this link https://www.crd.york.ac.uk/PROSPEROFILES/adf8616cfbe5f9edae93211c01f2fcff.pdf.

Selection process
Studies will be screened independently by at least two people (or person/machine combination) with a process to resolve differences.

Other relevant information about searching and screening
None

DATA COLLECTION PROCESS

Data extraction from published articles and reports
Data will be extracted by one person (or a machine) and checked by at least one other person (or machine).

Authors will not be contacted for further information.

Study risk of bias or quality assessment
Risk of bias will be assessed using: *Cochrane RoB-2*

Data will be assessed independently by at least two people (or person/machine combination) with a process to resolve differences.

Additional information will **not** be sought from study investigators if required information is unclear or unavailable in the study publications/reports.

Reporting bias assessment

Risk of bias due to missing results will be assessed

Certainty assessment

Certainty of findings will not be assessed

OUTCOMES TO BE ANALYSED

Main outcomes

1. Change in Spherical Equivalent Refraction (SER): Defined as the mean change in refractive error from baseline.

    1. Measurement instruments: Cycloplegic autorefraction or subjective refraction.
    2. Instruments: Data obtained via autorefraction (using an autorefractometer) or subjective refraction (using a phoropter/trial frame).
    3. Time points: Assessed at follow-up visits
    4. Effect measure: Mean Difference (MD) in diopters (D).

2. Change in Axial Length (AL): Defined as the mean change in ocular axial length from baseline.

    1. Measurement instruments: Optical biometry or A-scan ultrasonography.
    2. Time points: Assessed at follow-up visits.
    3. Effect measure: Mean Difference (MD) in millimeters (mm)

3. Myopia Incidence: Defined as the number of new myopia cases in prevention studies.

    1. Effect measure: Risk Ratio (RR) or Odds Ratio (OR).

Additional outcomes

1. Best Corrected Visual Acuity (BCVA): To assess safety and visual function.

    1. Measurement instruments: Standardized distance visual acuity charts (Snellen chart or Landolt C chart) at 6 meters (or 20 feet).
    2. Metric: Measured in logMAR lines or Snellen decimal equivalent.
2. Safety / Adverse events: Incidence of complications such as photophobia, dazzle, or ocular discomfort associated with light therapy or supplements.
3. Treatment adherence: Quantitative measurement of compliance with the intervention.

    1. Measurement method: Electronic device logs (for light therapy devices), pill counts (for supplements), or self-reported diaries/questionnaires.
    2. Effect measure: Percentage of compliant participants or mean percentage of completed sessions/doses.
4. Choroidal thickness : Change in subfoveal choroidal thickness (SFCT).

    1. Measurement instruments: Optical Coherence Tomography (OCT).
    2. Effect measure: Mean change measured in micrometers (μm

PLANNED DATA SYNTHESIS

Strategy for data synthesis

A standardized data extraction table will be created to facilitate the extraction of data from each included study.

1. If sufficient data are available, meta-analyses will be performed using the metan command in Stata statistical software (Stata v.17; StataCorp LLC, College Station, TX, USA).Continuous outcomes (SER, Axial Length): will be pooled using the Mean Difference (MD) with 95% Confidence Intervals (CI).

2. Dichotomous outcomes (Incidence, Adverse events): will be pooled using Risk Ratios (RR) or Odds Ratios (OR).
3. Heterogeneity: will be assessed using the I-squared (I^2) statistic. An I^2 value >50% will indicate substantial heterogeneity.
4. Model: A random-effects model will be employed for the meta-analysis due to the anticipated clinical and methodological diversity among studies.

After the systematic search, references will be exported to a reference management software to remove duplicates. Title and abstract screening will be conducted independently by two reviewers with expertise in pediatrics. Full-text screening will be performed independently by the same two reviewers, with discrepancies resolved by consensus or, when required, by a third reviewer.

Regarding data acquisition, study authors will not be contacted for further information or clarification. Data extraction and quality assessment will be based exclusively on the information available in the published articles and their supplementary materials. Furthermore, individual participant data (IPD) will not be sought; the synthesis of results will rely entirely on aggregate data (such as means, standard deviations, and sample sizes) provided in the study reports

CURRENT REVIEW STAGE

Stage of the review at this submission

Review stage	Started	Completed
Pilot work	✓	
Formal searching/study identification	✓	
Screening search results against inclusion criteria		
Data extraction or receipt of IPD		
Risk of bias/quality assessment		
Data synthesis		

Review status

The review is currently planned or ongoing.

Publication of review results

Results of the review will be published in English and Spanish.

REVIEW AFFILIATION, FUNDING AND PEER REVIEW

Review team members

Mrs Claudia Domínguez Morras (review guarantor and contact) ORCID: 0000-0002-8585-6400. UPNA. Spain.

No conflict of interest declared.

Dr Laura Moreno. Hospital Universitario de Navarra. Spain.

No conflict of interest declared.

Dr Miren Paniagua. Hospital Universitario de Navarra. Spain.

No conflict of interest declared.

Isabel Castro-Garrido. ORCID: 0009-0005-5605-2509. Hospital Universitario de Navarra. Spain.

No conflict of interest declared.

Dr Alejandro Fernández-Montero. Clínica Universidad de Navarra. Spain.

No conflict of interest declared.

Named contact

Mrs Claudia Domínguez Morras (dominguez.140576@e.unavarra.es). ORCID: 0000-0002-8585-6400. UPNA. Spain.

Review affiliation

Hospital Universitario de Navarra and Universidad Pública de Navarra.

Funding source

Review has no funding and no agreed support from an academic institution and is done in authors' own time.

Peer review

The protocol has been developed and internally peer-reviewed by the review team members at the Hospital Universitario de Navarra. Frequent meetings were held to refine the research question (PICO), eligibility criteria, and data extraction methods, ensuring the protocol meets methodological standards and alignment with PRISMA-P guidelines before registration

ADDITIONAL INFORMATION

Additional information

This protocol follows the PRISMA-P guidelines for systematic reviews

Review conflict of interest

Declared individual interests are recorded under team member details.. No additional interests are recorded for this review.

Medical Subject Headings

Myopia; Adolescent; Child; Anthocyanins; Dietary Supplements; Fatty Acids, Omega-3; Humans; Lutein; Minerals; Vitamins; Zeaxanthins; Micronutrients; Low-Level Light Therapy

PROSPERO version history

No preview available

Disclaimer

The content of this record displays the information provided by the review team. PROSPERO does not peer review registration records or endorse their content.

PROSPERO accepts and posts the information provided in good faith; responsibility for record content rests with the review team. The guarantor for this record has affirmed that the information provided is truthful and that they understand that deliberate provision of inaccurate information may be construed as scientific misconduct.

PROSPERO does not accept any liability for the content provided in this record or for its use. Readers use the information provided in this record at their own risk.

Any enquiries about the record should be referred to the named review contact
