# Supplementary material for: Non-Pharmacological and Non-Optical Interventions for Myopia Prevention and Control in Children: A Systematic Review and Meta-Analysis
Source: Children (Basel). 2026 Jul 10;13(7):915. doi: 10.3390/children13070915 (PMC13406435; doi:10.3390/children13070915)
Supplement: Supplementary file 1 [file children-13-00915-s001.zip › Supplementary File S4. RoB2_Assessment- non-pharmacological.pdf]

SUPPLEMENTARY MATERIAL:

RoB 2.0 Risk of Bias Assessment for Included RCTs

Impact of non-pharmacological interventions on myopia incidence and progression in children: a systematic review and meta-analysis

Abbreviations used in table are based on the individual domains assessed using the Cochrane Risk of Bias tool: the RoB 2 tool.

1. Randomization Process → Random.
2. Deviations from Intended Interventions → Deviations
3. Missing Outcome Data → Miss. Data
4. Measurement of the Outcome → Outcome
5. Selection of Reported Results → selection of RR
6. Overall Bias Judgment → Overall

Nutritional Supplementation

Study (Author, Year)	Random.	Deviations	Missing Data	Outcome	Selection of RR	Overall
**Klopotskaya N, el al (2020)**	High Risk	High Risk	High Risk	Some Concerns	High Risk	High Risk
**Omar, et al (2018)**	High Risk	High Risk	High Risk	Some Concerns	Some Concerns	High Risk
**Mori, et al. (2019)**	Low Risk	Low Risk	Low Risk	Low Risk	Low Risk	Low Risk
**Li et al. (2025)**	Low Risk	Low Risk	Low Risk	Low Risk	Low Risk	Low Risk

Repeated Low-Level Red-Light (RLRL) Therapy

**Study (Author, Year)**	**Random.**	**Deviations**	**Missing Data**	**Outcome**	**Selection of RR**	**Overall**
**Cao, et al. (2024).**	Low Risk	Some Concerns	Low Risk	Low Risk	Low Risk	Some Concerns
**Chen, et al. (2023).**	Low Risk	Some Concerns	Low Risk	Low Risk	Low Risk	Some Concerns
**Deen, et al. (2024)**	Low Risk	Some Concerns	Low Risk	Low Risk	Low Risk	Some Concerns
**Dong, et al. (2023).**	Low Risk	Low Risk	Low Risk	Low Risk	Low Risk	Low Risk
**He, et al. (2023)**	Low Risk	Some Concerns	Low Risk	Low Risk	Low Risk	Some Concerns
**Jiang, et al. (2022)**	Low Risk	Some Concerns	Low Risk	Low Risk	Low Risk	Some Concerns
**Liu G, et al. (2024)**	Low Risk	Some Concerns	Low Risk	Low Risk	Low Risk	Some Concerns
**Liu G, et al. (2025)**	Low Risk	Some Concerns	Low Risk	Low Risk	Low Risk	Some Concerns
**Liu G, et al. (2025)**	Low Risk	Some Concerns	Low Risk	Low Risk	Low Risk	Some Concerns
**Liu Z, et al. (2025)**	Low Risk	Some Concerns	Low Risk	Low Risk	Low Risk	Some Concerns
**Xiong, et al. (2024)**	Low Risk	Some Concerns	Low Risk	Low Risk	Low Risk	Some Concerns
**Xu, et al. (2024)**	Low Risk	Some Concerns	Low Risk	Low Risk	Low Risk	Some Concerns
**Zhou L, et al (2023)**	Low Risk	Some Concerns	Some Concerns	Low Risk	Low Risk	Some Concerns
**Zhou W, et al (2024)**	Low Risk	Some Concerns	Low Risk	Low Risk	Low Risk	Some Concerns

Table including the full title of each study, the overall RoB 2 risk rating, and a brief justification for the assessment provided. Nutritional Supplementation

**Study (Author, Year, Title)**	**Overall ROB 2-Bias Judgment**	**Justification**
Klopotskaya N, el al (2020); Selenium-Containing Antioxidant Therapy Eff ect on the Treatment Results in Children with Myopia, Depending on the State of Autonomic Nervous System	High Risk	No mention of randomization, vast disparities in treatment protocols between groups without placebo, and no reporting of dropout rates.
Omar, et al (2018); Effect of bilberry extract on slowing high-myopia progression in children: 2-year follow-up study	High Risk	Lack of details on randomization, no blinding/placebo, and omission of the dropout rate in the statistical results.
Mori, et al. (2019); The Effect of Dietary Supplementation of Crocetin for Myopia Control in Children: A Randomized Clinical Trial	Low Risk	Excellent double-blind design with an identical placebo, perfect allocation concealment, and retention rates over 97%.
Li et al. (2025); Effect of Lutein Ester Supplement on Choroidal Thickness in Children: A Randomized Controlled Trial	Low Risk	Rigorous double-blind trial with a placebo pill, masked evaluators, and appropriate statistical handling (ITT) of minimal missing data

Table including the full title of each study, the overall RoB 2 risk rating, and a brief justification for the assessment provided. Repeated Low-Level Red-Light (RLRL) Therapy

Cao, et al. (2024); Daily Low-Level Red Light for Spherical Equivalent Error and Axial Length in Children with Myopia: A Randomized Clinical Trial	Some Concerns	Excellent methodology, randomization, and measurement, but limited by the inability to blind families due to the lack of a sham device.
Chen, et al. (2023); Low-intensity red-light therapy in slowing myopic progression and the rebound effect after its cessation in Chinese children: a randomized controlled trial	Some Concerns	Adequate randomization and retention with masked measurement, but the open-label design (no placebo) introduces a risk of deviations from intended interventions.
Deen, et al. (2024); Three-Month Interim Analyses of Repeated Low-Level Red-Light Therapy in Myopia Control in Schoolchildren: A Multi-Ethnic Randomized Controlled Trial	Some Concerns	Rigorous allocation concealment and masked evaluators. Risk is limited to the absence of an inactive device to blind the control group.
Dong, et al. (2023); Myopia Control Effect of Repeated Low-Level Red-Light Therapy in Chinese Children: A Randomized, Double-Blind, Controlled Clinical Trial	Low Risk	A model trial and true double-blind. Succeeded in blinding patients and researchers using a sham device at 10% power.
He, et al. (2023); Effect of Repeated Low-level Red Light on Myopia Prevention Among Children in China With Premyopia: A Randomized Clinical Trial	Some Concerns	High sample retention and objective masked measurement. Risk of bias stems from the families' knowledge of the assigned treatment.
Jiang, et al. (2022); Effect of Repeated Low-Level Red-Light Therapy for Myopia Control in Children: A Multicenter Randomized Controlled Trial	Some Concerns	Multicenter trial with robust ITT analysis and masked evaluators, but a "single-blind" design that fails to mask the intervention from participants.
Liu G, et al. (2024); Effectiveness of repeated low-level red light in myopia prevention and myopia contro	Some Concerns	Proper subgroup analysis and objective measurement, but the control group did not use a sham device, preventing patient blinding
Liu G, et al. (2025); Two-year outcomes of repeated red light therapy in premyopic children: sustained efficacy and rebound effects	Some Concerns	Highly rigorous long-term follow-up. Penalized only by the lack of a placebo device to mask children and parents.
Liu G, et al. (2025); Axial Shortening Effects of Repeated Low-level Red-light Therapy in Children With High Myopia: A Multicenter Randomized Controlled Trial	Some Concerns	Excellent centralized randomization. Risk is concentrated in the deviations domain as it is a single-blind study (patients not blinded).
Liu Z, et al. (2025); The Effects of Repeated Low-Level Red-Light Therapy on the Structure and Vasculature of the Choroid and Retina in Children with Premyopia	Some Concerns	High-precision anatomical measurements (OCT) by masked technicians. However, allocation could not be concealed from the children's families
Xiong, et al. (2024); Effectiveness of low-level red light for controlling progression of Myopia in children and adolescents	Some Concerns	Perfect retention at 6 months (0% dropouts) and transparent data, but features an open-label intervention design for participants.
Xu, et al. (2024); Repeated Low-Level Red Light Therapy for Myopia Control in High Myopia Children and Adolescents: A Randomized Clinical Trial	Some Concerns	Flawless prospective registration and masked evaluators. As usual for this therapy, it suffers from a lack of participant blinding.
Zhou L, et al (2023); Photobiomodulation therapy retarded axial length growth in children with myopia: evidence from a 12-month randomized controlled trial evidence	Some Concerns	Added to the usual lack of blinding due to no placebo device, there is a 22% dropout rate that raises some reservations.

Zhou W, et al (2024); Efficacy of Different Powers of Low-Level Red Light in Children for Myopia Control	Some Concerns	Perfect randomization into 4 groups and minimal missing data. The only risk domain arises from lacking a sham device.
